# Attitudes of potential recipients toward emerging visual prosthesis technologies

**DOI:** 10.1038/s41598-023-36913-8

**Published:** 2023-07-06

**Authors:** Vicky Karadima, Elizabeth A. Pezaris, John S. Pezaris

**Affiliations:** 1 Multisensory and Temporal Processing Lab (MultiTimeLab), Department of Psychology, Panteion University of Social and Political Sciences, Athens, Greece; 2 Department of Psychology, Boston College, Boston, MA USA; 3 Department of Neurosurgery, Massachusetts General Hospital, Boston, MA USA; 4 Department of Neurosurgery, Harvard Medical School, Boston, MA USA

**Keywords:** Human behaviour, Macular degeneration, Visual system, Eye diseases

## Abstract

With the advent of multiple visual prosthesis devices to treat blindness, the question of how potential patients view such interventions becomes important in order to understand the levels of expectation and acceptance, and the perceived risk-reward balance across the different device approaches. Building on previous work on single device approaches done with blind individuals in Chicago and Detroit, USA, Melbourne, Australia, and Bejing, China, we investigated attitudes in blind individuals in Athens, Greece with coverage expanded to three of the contemporary approaches, Retinal, Thalamic, and Cortical. We presented an informational lecture on the approaches, had potential participants fill out a preliminary Questionnaire 1, then organized selected subjects into focus groups for guided discussion on visual prostheses, and finally had these subjects fill out a more detailed Questionnaire 2. We report here the first quantitative data that compares multiple prosthesis approaches. Our primary findings are that for these potential patients, perceived risk continues to outweigh perceived benefits, with the Retinal approach having the least negative overall impression and the Cortical approach the most negative. Concerns about the quality of restored vision were primary. Factors that drove the choice of hypothetical participation in a clinical trial were age and years of blindness. Secondary factors focused on positive clinical outcomes. The focus groups served to swing the impressions of each approach from neutrality toward the extremes of a Likert scale, and shifted the overall willingness to participate in a clinical trial from neutral to negative. These results, coupled with informal assessment of audience questions after the informational lecture, suggest that a substantial improvement in performance over currently available devices will be necessary before visual prostheses gain wide acceptance.

## Introduction

The purpose of this study is to understand the expectations and attitudes of blind and partially blind persons who are interested in restoring lost visual function through visual prostheses. Such devices use artificial means to convert patterns of light into neural signals and deliver them to the visual system when the natural biological structures of the eyes are no longer able to do so. Although the ideas behind visual prostheses have been described in science fiction writing for decades, and experiments performed for nearly 100 years (see historical reviews by Donaldson and Brindley^1^, and Lewis and Rosenfeld^2^), only recently has technological advancement lead to promising successes with human implantation and experimentation^3–11^. The field as a whole is still in its infancy and restored sight remains crude^12–14^, although the future holds promise for improved results^15–17^.

Here, we cover three of the current approaches to visual prosthesis devices, *retinal*, *thalamic*, and *cortical*, to expand upon previous studies that concentrate on single devices^18–23^, and make comparisons between approaches with regard to how they are perceived by their target population. Our results will help guide the design of patient selection criteria and inform future prosthesis device development.

### Target population

The target population for visual prostheses includes previously sighted individuals who have lost their sensitivity to light due to a variety of factors, including disease and trauma. Typical patients are in the last stages of vision loss from diseases like glaucoma, retinitis pigmentosa or macular degeneration, with moderate to severe visual impairment.

#### Effects of vision impairment

Acquired visual impairment has wide-reaching impact on health, social, and economic status. It reduces quality of life and increases the risk of death^24,25^. Common aspects of normal life, including mobility, domestic activities, interpersonal interactions and relationships, economic transactions, and social and civic-related activities, become challenging with visual impairment^26,27^. Visual impairment can influence depression, anxiety^28^, cognitive functioning^29,30^, create loss of well-being^31,32^, and increase risk of suicide^33,34^. The loss of visual function creates economic burdens on the individual and the state related to costs of treatment, services, devices, and lost earnings caused by reduced productivity^35–37^.

Rehabilitation needs reported by visually impaired persons can be informative of challenges for those living with low vision and the resiliencies available to them^38,39^. Among the most crucial concerns of people with visual impairment are possible treatments including vision restoration^40^.

#### Treatment with visual prostheses

While treatment with a visual prosthesis meshes well with these desires for vision restoration from the potential patient population, some caution is due as patient expectations may not match well with results available from clinical or even experimental devices. Special attention needs to be paid to patient attitudes toward visual enhancement^41–44^. For the purposes of this paper, we will consider three potential treatment approaches, as described in the following paragraphs.

### Three visual prosthesis approaches

Visual prosthesis devices typically include a digital camera to capture the visual scene, electronic processing to translate the video images into the neural code, and a means to deliver translated signals directly to the early stages of the visual system. Although the resolution of restored vision is quite low^4,6,14,45^, it is thought to be beneficial^9,46,47^ with some caveats^11,48–50^. Devices are classified by which stage of the visual pathway they target for delivery of visual information, with the three approaches addressed here being the retina (thus the *retinal approach*), the lateral geniculate nucleus of the thalamus (*thalamic approach*) and the primary visual cortex (*cortical approach).* Each will be briefly summarized below, while the reader is directed to the many contemporary reviews for more detail (e.g.^6–8,51^).

#### Retinal approach

Retinal approaches deliver visual information through electrical stimulation of the retina. These devices require less invasive implantation than other approaches, and have the potential to provide representation of the full visual field, although no current device does^51^. Only a handful of such devices have been approved for clinical testing or use, worldwide, including Argus II (Second Sight Medical Products, USA)^41,52^, Alpha IMS (Retina Implant AG, Germany)^53,54^, BVT Bionic Eye System (Bionic Vision Technologies, Australia)^55^, Iris II and PRIMA (Pixium Vision, France)^56^, EPIRET3 (Epi-Ret GmBH, Germany)^57^, but there continues to be substantial pre-clinical and clinical research^6–8^. As of this report, these devices have been approved only for treatment of retinitis pigmentosa, although they should be applicable to early stages of macular degeneration and a number of less common diseases as well.

#### Thalamic approach

The thalamic approach^58^ delivers visual information through electrical stimulation of the lateral geniculate nucleus of the thalamus (LGN). These devices are in pre-clinical development with ongoing work using both animal^58,59^ and human models^60–64^. It is anticipated that although implantation will require brain surgery, it will be minimally invasive^51,58^. The approach relies on established stimulation technology and applies to a wide range of diseases including glaucoma, macular degeneration, diabetic retinopathy, or trauma to the early visual pathway^51^.

#### Cortical approach

The cortical approach^65^ delivers visual information through electrical stimulation of the visual cortical areas located at the rear of the head^7,8,14^. As the visual cortex is a large area, it is ideal for implantation of a large number of electrodes that would provide higher visual resolution and potentially restoration of more visual functions^51^. The cortical approach requires the most invasive surgery, as typically a large skull opening is required to place electrode arrays, and faces substantial challenges to place electrodes in the important foveal area^48^. Nevertheless, significant work continues in both pre-clinical^16^ and clinical levels^66,67^. The cortical approach applies to the same wide range of disease conditions as the thalamic approach.

### Structure and purpose of this study

Previous studies have concentrated on the views of potential patients toward a single device, leaving open the larger picture of views toward visual prostheses in general. Here, we address that question by investigating attitudes candidates express if they could choose among the three basic approaches to artificial vision.

We gathered information on subjects’ perspectives for implantation as well as participants’ hopes, fears, and their perceived risk/benefit balance. Other issues we were interested in understanding were the sources of information that potential candidates used, and the primary factors that might drive their decisions.

In order to explore these questions, we used two basic tools, questionnaires and focus groups, recruiting blind and low-vision individuals to provide their opinions and views on the subject of visual prostheses and sight restoration. In this report, we present the results from the two questionnaires that were filled out before and after the focus group sessions, respectively.

#### Focus groups and questionnaires

Focus groups can be used to collect information about the characteristics of treatment candidates, as well as to help candidates review risks and benefits before consenting to clinical trials. Focus groups in artificial vision usually include presentation of potential treatment options, as well as an explanation of the specific device the research team is developing^20^. Focus group discussions include clarification of poorly understood information, review of opinions, and exploration of attitudes and motivation of each candidate.

Complementing focus groups, individual interviews of structured or semi-structured questionnaires help researchers examine themes relevant to each participants’ motivation, their decision factors and expectations. Such data aid the development of materials for informed consent, and may be used to help identify candidates for clinical trials^18^.

### Cooperation with *Faros Tyflon*

This study was performed with the cooperation of *Faros Tyflon* (Φάρος Τυφλών Ελλάδας, The Lighthouse for the Blind of Greece) in Athens, Greece, a charitable institution devoted to supporting the visually impaired Greek community. Their members are Greek nationals, and the various aspects of this study were conducted in the Greek language. Importantly, no artificial vision technology was being tested or offered in Greece at the time of data collection.

## Results

We invited visually impaired people to participate in questionnaires covering topics around artificial vision, both before and after focus groups on the same subject. The questionnaires were based on those by Xia and colleagues^21^, expanded to include all three approaches to visual prostheses: retinal, thalamic, and cortical. The focus groups were based on the interview approach of Lane and colleagues^18,19,68^, again expanded to cover all three prosthesis types. Here we report on the quantitative results from the two questionnaires; the focus group results will be reported separately.

For analyses of the two questionnaires, we concentrated on three aspects of the quantitative results: participant demographics (see Section “Subject demographics”), the attitudes toward each approach and changes that appeared as a result of focus group participation (see Section “Attitudes toward the three approaches and how they changed from Q1 to Q2”), and the explanatory power of certain factors for key answers (see Section “Explanatory powers”).

In the following sections, we will present questions in capitalized, titular form (e.g., “Retinal Benefit” or “Education”), subject answers or selections in italics (e.g., “*unsure*”, or “*face perception*”) and combinations of the two separated by colons (e.g., “Choice Reasons: *surgical intervention*”, or “Information Sources: *services for the blind*”).

### Subject demographics

Subjects were recruited from the audience members of a presentation given by the authors at *Faros Tyflon.* The presentation reviewed the three main approaches to visual prostheses, retinal, thalamic and cortical and their state of the artS1. Immediately following the presentation, audience members were invited to fill out *Questionnaire 1: Short* (Q1 S1, S1). From the respondents, a collection of focus groups were conducted S2 to discuss the presentation. Once the focus groups concluded, those participants were asked to fill out *Questionnaire 2: Long* (Q2 S3).

**Figure 1:**
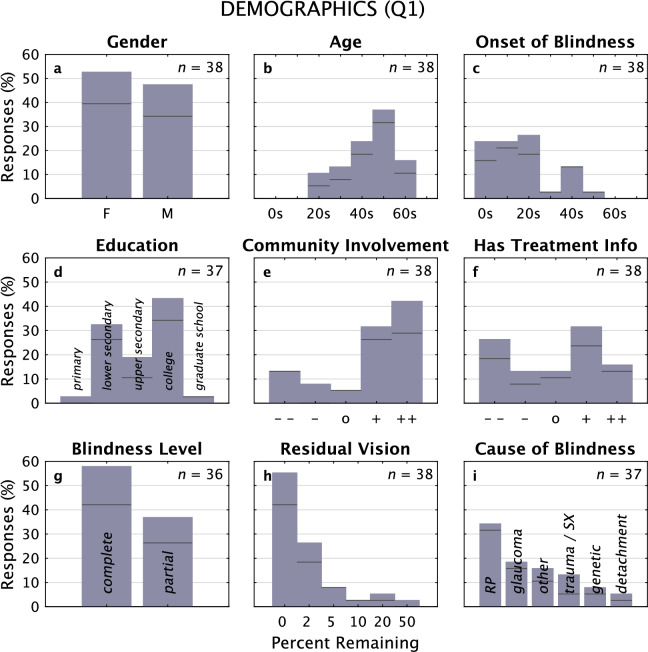
Gender, Age, Onset Age, Education, Community, Info. Responses for demographic questions in Questionnaire 1: Short are shown as frequency bar graphs. Unless indicated otherwise, the number of responses is 38. For Age and Onset of Blindness, values were grouped into decades of life. Responses from the subset of subjects who also completed Questionnaire 2: Long are shown with black horizontal lines on each bar. No statistically significant differences were found between responses for subjects who only completed Q1 (Q1-only, *n* = 10) and those who went on to participate in a focus group and then complete Q2 using Wilcoxon rank sum comparisons with a threshold of *p* = 0.05 (the range of observed *p* values was 0.1 to 0.8), except for Onset Age (*p* = 0.01), which skewed to a younger range for Q1-only participants, and Cause of Blindness (*p* = 0.02), which was more distributed to uncommon causes for Q1-only participants.

A total of 38 individuals completed Q1 (Fig. 1; two additional individuals gave only their contact information and are not included in this report; occasional questions were not answered by individual participants, so the reported counts are sometimes slightly less than 38). There were 21 F/17 M subjects; ages ranged from 25 to 67 with the plurality in their 50 s. Most subjects (76%, 29/38) had an onset of blindness before 30 years old. Education levels were across the board, whereas involvement in the *Faros Tyflon* community was at the upper end of the Likert scale (74%, 28/38 at + or ++; see “Methods” for Likert encoding). Slightly over half (58%, 22/36) described their impairment as complete rather than partial, which was confirmed when they reported quantitative levels of residual vision. The two primary causes of blindness for this group were *retinitis pigmentosa* (RP) and *glaucoma*, with a combined fraction of 54% (20/37); the balance was spread among a wide range of causes of comparatively low frequency. The distribution of distances from each subject's home to the heart of Athens where the presentation took place was consistent with a log-linear layout (*R*^2^ = 0.7) as expected for populations surrounding European urban centers^69^.

A subset of subjects who completed Q1 volunteered to continue on with the focus group process and then completed Q2 (*n* = 28, 15 F/13 M); the remaining subset voluntarily withdrew from the study. To ensure the continuing-on subgroup was representative of the larger set, we compared Q1 responses from the two sub-groups (those who stopped after Q1, or *Q1-only*, and those who continued on, or *Q1&2*) and found no statistically significant differences for any demographic question (Wilcoxon rank sum, *p* = 0.1 or higher on each test) except for Onset Age and Cause of Blindness. For Onset Age, the distributions are significantly different (Wilcoxon rank sum, *p* = 0.01) with the set of Q1-only subjects being blind from an earlier age than the set of Q1&2 subjects. For Cause of Blindness, the Q1-only sub-group had a wider range of etiologies than the Q1&2 sub-group (*p* = 0.02). For the remainder of this presentation, we will examine results only for the 28 subjects in Q1&2 who completed both questionnaires (and, therefore, also went through the focus group experience), except where indicated. One of the Q1&2 subjects partially completed Q2, thus many of the results reported below show a count of 27 rather than 28. In other cases, some subjects chose not to respond to individual questions and the count is sometimes lower.

### Attitudes toward the three approaches and how they changed from Q1 to Q2

To understand if the focus group experience itself would change subjects’ viewpoints, we compared responses before and after for items that were posed identically on the two surveys. As a control, we verified that the basic demographic information was consistent between questionnaires for individual subjects (mean and standard deviation of paired differences of Age, 0.0 ± 0.0, Wilcoxon signed rank *p* = 1.0; Education, 0.04 ± 0.19,* p* = 1.0; Onset Age, –0.18 ± 0.67, *p* = 0.5; Residual Vision, 0.00 ± 0.01, *p* = 0.8; distance from town or neighborhood of Residence to center of Athens, 0.00 ± 0.00, *p* = 1.0). For other questions, we looked for changes. In particular, we were concerned that the distribution of Has Treatment Information might have changed as a result of the focus groups, but there was no difference (Wilcoxon signed rank, *p* = 0.7). Upon closer examination, we found that the mean score improved only very slightly (mean and standard deviation of paired differences, 0.07 ± 1.14), with the majority (16 subjects) not changing, five scores going up and six going down, suggesting that the focus group experience itself did not inherently provide additional information (but see below for changes in attitudes).

#### Benefit vs risk

**Figure 2:**
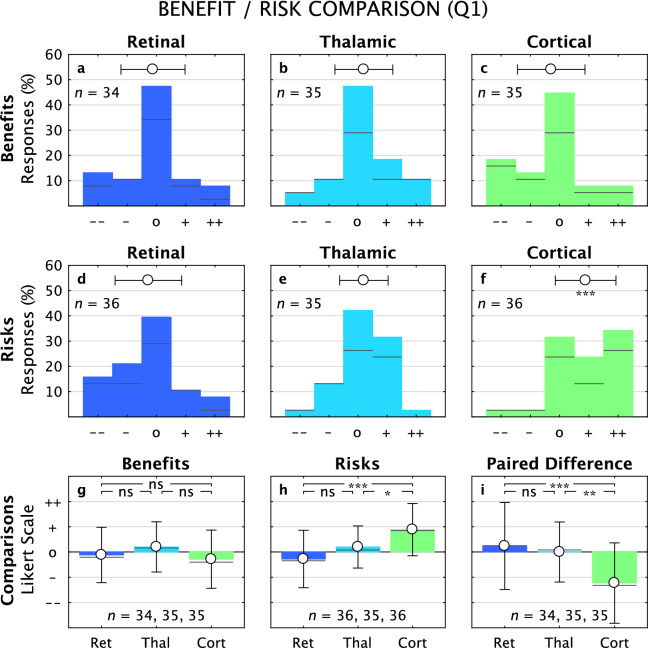
Benefit/Risk, Questionnaire 1. Distribution of Likert scale responses to Benefits and Risks questions from Q1 are shown across the three approaches, along with comparisons between approaches (Retinal, dark blue; Thalamic, light blue; Cortical, green; these same colors are used in subsequent figures). For Panels (**a**–**f**), black marks separate responses from Q1&2 (below line) to those from Q1-only (above line); for Panels (**g**–**i**), the black marks represent the mean values for Q1&2 data alone, and the colored bars for data from both Q1-only and Q1&2. The distribution of Benefits (**a**–**c**) was not significantly different from the neutral response for all three approaches. The distribution of Risks (**d**–**f**) was not significantly different from neutral for Retinal and Thalamic approaches, but was significantly positive for the Cortical approach. When comparing Benefits across approaches (**g**), no significant differences were found. For Risks (**h**), significant differences were found for all comparisons. For the Paired Difference (or Net Benefit, computed as Benefit minus Risk), Retinal and Thalamic approaches were not significantly different from neutral, and significant differences were found only with comparisons to the Cortical approach, which was significantly negative.

The central questions we asked concerned the perceived benefits and risks associated with each of the three approaches. In general, the focus groups served to amplify views away from neutral for Benefit/Risk balances; we will first review Q1 responses and then describe those from Q2 in the following paragraph. For Q1 (Fig. 2), Benefits for each of the three approaches were dominated by the neutral response (Retinal –0.1 ± 1.1, *p* = 0.5, *n* = 34; Thalamic 0.2 ± 1.0, *p* = 0.2, *n* = 35; Cortical –0.3 ± 1.2, *p* = 0.2, *n* = 35, *t*-tests in all cases) and none of the distributions had means that significantly deviated from that value (Fig. 2a–c), while the Risks were lowest for Retinal (–0.3 ± 1.1, *p* = 0.15, *n* = 36), higher for Thalamic (0.2 ± 0.8, *p* = 0.16, *n* = 35), and even higher for Cortical (0.9 ± 1.0, *p* = 0.001, *n* = 36) with the means deviating significantly from neutral only for the Cortical responses (Fig. 2d–f). While there were no significant differences for pairwise comparisons of the distributions of Benefits (ANOVA, *F*_2, 101_ = 1.8, *p* = 0.17; *p*_*R-T*_ = 0.4, *p*_*R-C*_ = 0.8, *p*_*T-C*_ = 0.2; Fig. 2g), pairwise comparisons for the distributions of Risks reflected significant increases along the Retina-Thalamic-Cortical chain (ANOVA,* F*_2, 104_ = 12,* p* = 0.000; *p*_*R-T*_ = 0.1, *p*_*R-C*_ = 0.000, *p*_*T-C*_ = 0.01; Fig. 2h). The net benefit, or per-subject paired difference between Benefit and Risk scores, showed qualitative similarity between Retinal (0.2 ± 1.7, *t*-test *p* = 0.4, *n* = 34) and Thalamic (0.0 ± 1.2, *t*-test *p* = 1.0, *n* = 35) approaches but a substantially negative net score for the Cortical approach (–1.2 ± 1.6, *t*-test *p* = 0.001, *n* = 35). That distinction was emphasized by pairwise examination between approaches; the difference between Retinal and Thalamic distributions not rising to significance, but both comparisons with the Cortical distribution were highly significant (ANOVA, *F*_2, 101_ = 9.3,* p* = 0.000; *p*_*R-T*_ = 0.8, *p*_*R-C*_ = 0.000, *p*_*T-C*_ = 0.003; Fig. 2i).

**Figure 3:**
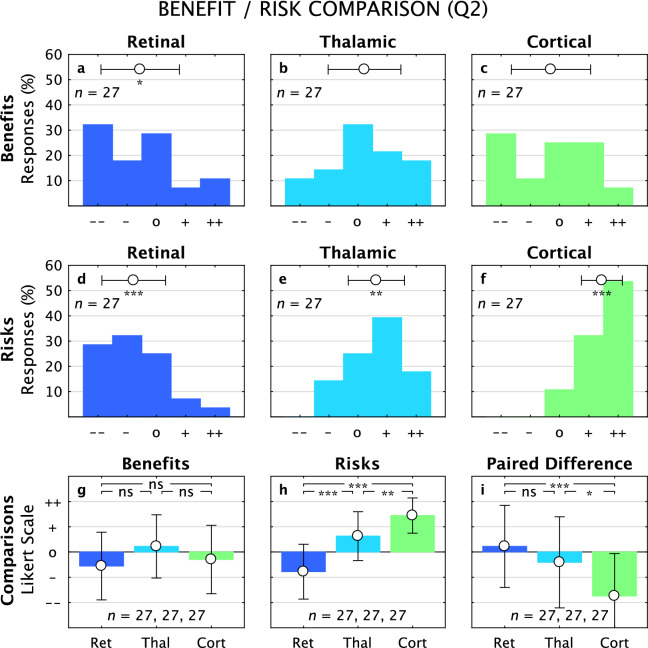
Benefit/Risk, Questionnaire 2. Distribution of Likert scale responses to Benefits and Risks questions from Q2 are shown for the three approaches (colors as before). Benefits are shown in the top row (**a**–**c**), with only the Retinal approach significantly different from neutral. Risks are shown in the middle row (**d**–**f**), with all three deviating significantly from neutral. Comparisons between the approaches are shown in the bottom row (**g**–**i**). For Benefits (**g**), there is a significant difference between Retinal and Thalamic approaches, but not for the other pairs. For Risks (**h**), there are significant differences between all pairs. For the Paired Difference (Benefit minus Risk), only the Cortical value was significantly different from zero and from the others.

For Q2, after the focus group, the Benefit/Risk distributions reflected substantial changes that moved the population away from neutrality and toward one extreme or the other (Fig. 3). For all three cases, the Benefits distributions flattened (Fig. 3a–c), with the Retinal distribution becoming significantly below neutral (–0.6 ± 1.3, *p* = 0.04, *n* = 27), the Thalamic distribution remaining indistinguishable from neutral (0.2 ± 1.3, *p* = 0.4, *n* = 27), and the Cortical distribution also indistinguishable from neutral (–0.3 ± 1.4, *p* = 0.3, *n* = 27). For Risks, the trend seen in Q1 responses were amplified and all three distributions moved, becoming significantly deviated from neutral as the perceived risk for the Retinal approach dropped (–0.8 ± 1.1, *p* = 0.001, *n* = 27), and the risks for both Thalamic (0.6 ± 1.0, *p* = 0.002, *n* = 27) and Cortical (1.4 ± 0.7, *p* = 0.001, *n* = 27) approaches increased (Fig. 3d–f); notably, 89% of the responses (24/27) were above neutral for Cortical Risk. Pairwise comparisons of Benefits between approaches revealed a difference between Retinal and Thalamic approaches that did not quite rise to significance, and no significances between the other two pairs (ANOVA, *F*_2,78_ = 2.4, *p* = 0.09; *p*_*R-T*_ = 0.08, *p*_*R-C*_ = 0.7, *p*_*T-C*_ = 0.3; Fig. 3g). The profiles for Risks, however, became amplified, with Retinal decreasing (–0.8 ± 1.1, *p* = 0.001, *n* = 27) and both Thalamic (0.6 ± 1.0, *p* = 0.002, *n* = 27) and Cortical (1.4 ± 0.7, *p* = 0.000, *n* = 27) increasing to form a striking staircase, and significant differences all around (ANOVA, *F*_2, 78_ = 39, *p* = 0.000; *p*_*R-T*_ = 0.000, *p*_*R-C*_ = 0.000, *p*_*T-C*_ = 0.005; Fig. 3h). The net benefit (Benefit minus Risk) reflected this amplification with the Retinal approach remaining a net positive (0.2 ± 1.6, *t*-test *p* = 0.48, *n* = 27), the Thalamic approach a net negative (–0.4 ± 1.8, *t*-test *p* = 0.25, *n* = 27), and the Cortical approach a strong net negative (–1.7 ± 1.7, *t*-test *p* = 0.001, *n* = 27) with expected comparison significances (ANOVA, *F*_2, 78_ = 9.3, *p* = 0.000; *p*_*R-T*_ = 0.4, *p*_*R-C*_ = 0.000, *p*_*T-C*_ = 0.014; Fig. 3i).

#### Subject willingness to receive a visual prosthesis

**Figure 4:**
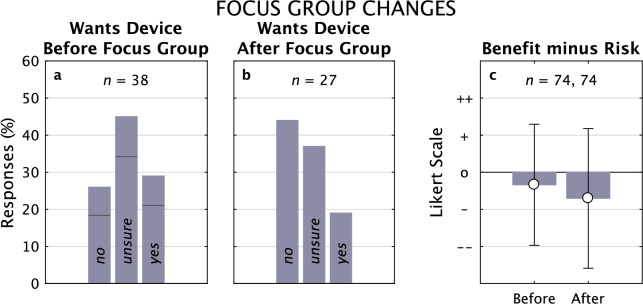
Wants Implant, Questionnaires 1 and 2. Responses to whether the subject would want a visual prosthesis implant before (**a**) and after (**b**) the focus group. From a predominantly neutral position in Q1, the population response moved substantially in the negative direction in Q2 after the focus group. This shift was reflected in the net benefit (Benefit minus Risk) aggregated for all approaches (**c**) which moved in the negative direction, albeit non-significantly. For Benefit minus Risk, some subjects gave partial responses on Q1, lowering the total count from an expected 81 responses (3 × 27) to 74 (24 + 25 + 25); while the matching responses were selected from Q2 here, using the full set does not appreciably change the result.

**Figure 5:**
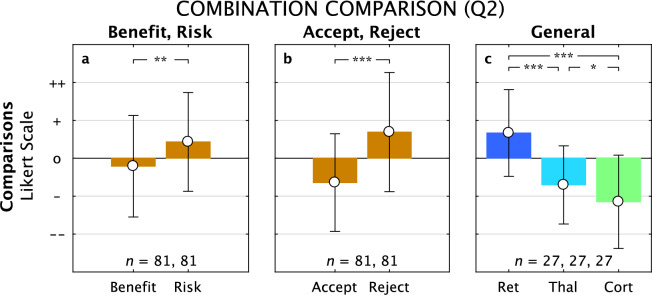
Comparisons for Benefit/Risk, Acceptance/Rejection, and General Attitude. Distribution of benefit and risk (**a**), and accept and reject (**b**) responses are shown pooled (orange) for the three approaches for Q2, along with distributions of the General Attitude responses for the three approaches (**c**, colors as in earlier figures). Significant differences were found for both Benefit versus Risk, with Risk significantly higher, and for Accept versus Reject, with Reject significantly higher. The General Attitude was positive for the Retinal approach, and became increasingly worse for the Thalamic and Cortical approaches, with significant differences in all pairs.

Perhaps the most telling change in responses before and after the focus groups was in whether subjects felt they wanted a visual prosthesis device (Fig. 4). Before the focus groups, 29% of subjects felt positively toward getting an implant; afterward, that number fell to 19%, and the number of subjects who felt negatively increased by a larger margin from 25 to 44%, with the additional negative responses coming from both the *Unsure* and *Yes* pools. A paired *t-*test analysis shows the responses, coding *No* as –1, *Unsure* as 0, and *Yes* as +1, shows a trend toward significance (*n* = 27 pairs, *p* = 0.069), and a bootstrap analysis shows the distributions are significantly different (*n* = 1000, *p* << 0.001). That strong movement was more weakly reflected in the calculated net benefit that was pooled across all approaches (Fig. 5). The net benefit was more negative after the focus groups than before, with a non-significant trend (*t*-test, *p* = 0.10), but a highly significant change with bootstrap (*n* = 1000, *p* << 0.001).

#### Most important ability for restoration

**Figure 6:**
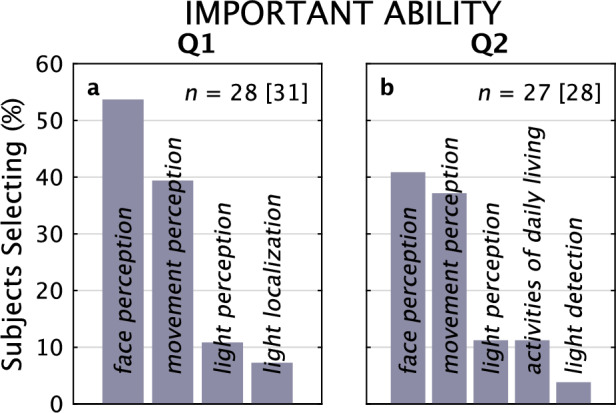
Important Abilities for Visual Prostheses to Support, Questionnaires 1 and 2. Responses from Q1 (**a**) and Q2 (**b**) are shown for the most Important Ability that subjects wanted from a visual prosthesis. Face Perception and Movement Perception dominated both distributions. For Q1 and Q2, 31 and 28 subjects made 28 and 27 selections, respectively.

We asked subjects to select which lost ability from a provided short list would be the most important for a visual prosthesis to restore (Fig. 6). This list was not exhaustive (see review by Dagnelie^70^ for a more in-depth exploration). Subjects responded consistently as a population before and after the focus groups that *face perception* (53% vs 39%, respectively) and *movement perception* (39% vs 36%) were the two most important abilities they would like restored, although many individual respondents switched between those two answers. The drop in *face perception* was primarily driven by the introduction of the additional option of *activities of daily living* in Q2, which siphoned off *face perception* responses to score at 11%. Tellingly, two write-in answers were, “I want my vision to be like it was before,” and, “To be able to go around on my own.”

### Motivations, concerns and aversions about participation in a clinical trial

**Figure 7:**
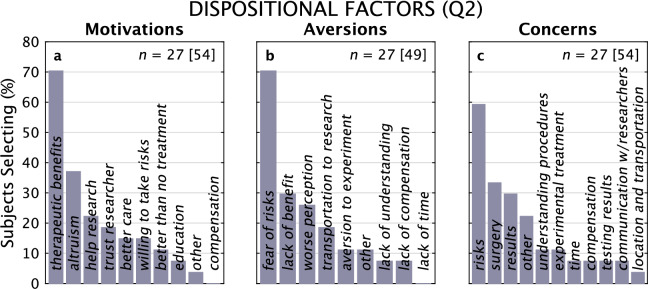
Motivations, Aversions and Concerns, Questionnaire 2. Distributions of Motivations for participating in a hypothetical clinical trial of an experimental device (**a**), Aversions to participation (**b**), and the Concerns expressed by subjects about participation (**c**) are shown for Q2. In both Aversions and Concerns, risk is the primary response. For all three questions, subjects were allowed to pick multiple options; the 27 responding subjects made 54, 49, and 54 selections, respectively.

We asked subjects to describe participating in a hypothetical clinical trial of a visual prosthesis and whom they might consult for advice regarding a decision to participate, making multiple selections from the list of possibilities for each question (Fig. 7). The question on Motivation was asked for both Q1 and Q2, and for Aversions and Concerns for Q2. The top three selections for Motivations were to obtain *therapeutic benefits*—that is, to be able to see again—(68%), *altruism* (36%), and to *help research* (21%). Interestingly, financial compensation was selected the least frequently (0%). These results were consistent between Q1 and Q2.

In Q2 (but not in Q1), we asked subjects to indicate their Aversions to participating in a trial and their Concerns about participation, again allowing multiple selections for both (Fig. 7). The primary Aversion was *fear of risk* (68%) followed by *lack of benefit* (29%) and *worse perception* (25%). The primary Concern was the *risk* associated with participation (57%) followed by *surgery* (32%), and *results* (29%).

#### Sources of advice and of information

**Figure 8:**
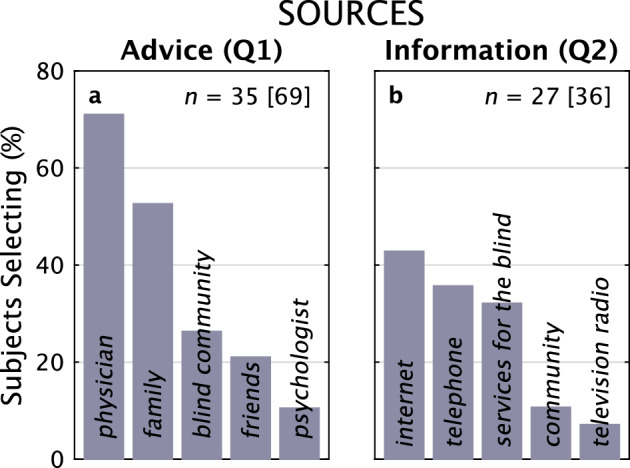
Sources for Advice and Information. The distribution of answers to where subjects would seek Advice about participation in a hypothetical clinical trial, asked in Q1, and what Information Sources they would use, asked in Q2. For Advice and Information Sources, 35 and 27 subjects made 69 and 36 selections, respectively.

Continuing on with questions about a hypothetical clinical trial, the two primary Advice Sources for making a decision to participate (Fig. 8) were the subject’s *physician* (71%) and *family* (53%). These results were consistent from Q1 to Q2, although the pattern of seeking advice primarily from physicians and less from other sources was amplified.

In Q2 (but not Q1), we asked subjects for their Information Sources (Fig. 8). As has become true for the rest of society, our subjects responded that the *internet* was their primary source (43%), followed closely by the *telephone* (36%), and *services for the blind* (32%).

#### Preferred approach

**Figure 9:**
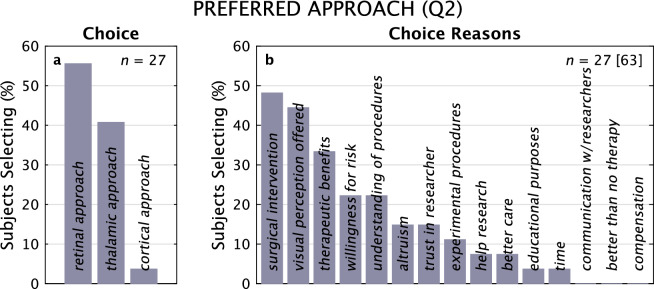
Preferred Approach, Reasons for Choice, Questionnaire 2. Distributions to answers of which device approach subjects would select, along with answers to why they made their choice are shown, from Q2. For both Choice and Choice Reasons, 27 subjects made 27 and 63 selections, respectively.

We then asked (in Q2) of the three approaches, which the subjects would prefer. Bearing in mind that an actual decision would include medical factors that might not have been considered by participants (e.g., retinal implants would likely not be applicable to patients with glaucoma), subjects selected devices (Fig. 9) as Retinal, 54%; Thalamic, 39%; Cortical, 4%, following a pattern reflective of the Benefit/Risk balance expressed by the population (Fig. 3i). The primary factors given for driving their choice were *surgery* (46%), the level of *visual perception offered* (43%), and the *therapeutic benefits* (32%).

Subjects were then asked in Q2 to rate acceptance and rejection levels for each device on Likert scales (Supplementary Fig. S2) followed by an overall impression (Supplementary Fig. S3). Acceptances were all negative (Retinal –0.5 ± 1.3, *p* = 0.045; Thalamic –0.4 ± 1.3, *p* = 0.15; Cortical –1.0 ± 1.2, *p* = 0.002; *n* = 27 in all cases) and Rejections were all positive (Retinal 0.3 ± 1.7, *p* = 0.4; Thalamic 0.6 ± 1.5, *p* = 0.044; Cortical 1.1 ± 1.4, *p* = 0.002; *n* = 27 in all cases). The net Accept/Reject responses qualitatively tracked those from Benefits/Risks (Figs. 2, 3, 5), with Retinal having the highest net rating (mean value –0.8 ± 2.5, *t*-test *p* = 0.1, *n* = 26), followed by Thalamic (–1.0 ± 2.6, *p* = 0.05, *n* = 22), and then Cortical approaches (–2.2 ± 2.2, *p* = 0.0001, *n* = 24), although none entered positive territory. The General Impression for each approach provided a succinct summary with the Retinal distribution positively deviated from neutrality (0.7 ± 1.1, *t*-test, *p* = 0.005, *n* = 20), and both the Thalamic (–0.5 ± 1.0, *p* = 0.002, *n* = 18) and Cortical (–1.1 ± 1.2, *p* = 0.0001, *n* = 25) distributions negatively deviated from neutrality; paired comparisons were all highly significant, indicating distinct responses.

### Explanatory powers

Correlations were run between answers to different questions to look for linkages to the primary measures of subject attitudes: Clinical Trial Participation, Benefit, Risk, Acceptance, Rejection, Choice (Preferred Approach). Results are presented in the following subsections starting with Clinical Trial Participation, the most important question of the study, followed by a summary analysis of the most influential factors on the remaining primary measures (Benefit, Risk, etc.).

#### Willingness to participate in a clinical trial

To understand the factors driving a subject toward their answer of the primary question, *would you be willing to participate in an experimental trial?,* we computed Spearman’s rank coefficients for the answers to each of the other questions. For Q1, two factors were significant: Years of Blindness, *r*_*s*_ = –0.48, *p* = 0.01, signifying that the longer a subject had been blind, the less likely they were to say “yes”; and Age of Onset, *r*_*s*_ = 0.50, *p* = 0.007, signifying subjects who were older when blinded would be more likely to say “yes”.

When we analyzed the responses to Q2 for the same question, the results were consistent: participation significantly correlated with Years of Blindness, *r*_*s*_ = –0.50, *p* = 0.008, and Age of Onset, *r*_*s*_ = 0.44, *p* = 0.02. The wider set of questions allowed for more detail as follows. Education became significant at *r*_*s*_ = –0.51, *p* = 0.006, indicating that after the focus group, subjects with higher education were now less inclined to hypothetical participation. Motivations: *therapeutic benefits* was significant at *r*_*s*_ = –0.45, *p* = 0.02, and Motivations: *better care* at *r*_*s*_ = 0.42, *p* = 0.03, together emphasizing the importance of clinical outcomes. Aversions: *transportation to research* was significant at *r*_*s*_ = 0.38, *p* = 0.048, reflective of the general difficulties faced by blind individuals in mobility. Finally, the related pair of Choice Reasons: *willingness for risk* with positive correlation at *r*_*s*_ = 0.40, *p* = 0.038, and Choice Reasons: *surgical intervention* with negative correlation at *r*_*s*_ = –0.44, *p* = 0.023 reflected two ends of a spectrum of attitudes regarding invasive treatments.

#### The most influential factors

We split the questions into two groups, those that could be considered demographic or subject introspective factors which we used as inputs to an influence analysis, and those that could be considered opinions on artificial vision which we used as outputs. The first group contained questions like age, educational level, sources of advice, motivations, aversions, etc. The second group contained questions on benefits, risks, acceptance, and rejection, etc. We computed Spearman rank correlations of the first group (inputs) against the second (outputs), and then used the resulting array of *p* values to sort the input factors by overall influence on each output factor. While a given input factor might be highly correlated with a particular output (as presented above), we wanted to identify input factors that were more frequently significant than others, overall, that thus could be considered fundamental influences on the formation of opinions. We used an index (*m*_*R*_) that was the mean across output factors of the ordinal position of each input factor when sorted by *p* value, normalized by the number of input factors. The values of *m*_*R*_ thus ranged from 0 to 1; the lower the value of *m*_*R*_, the more often a factor had a lower-ranked* p* value from a Spearman Rank correlation, and thus the more influence it wielded. Note that while *m*_*R*_ was based on the significance of each correlation, the strength of the correlation, including whether positive or negative, was not considered when computing the index.

Using this method, we found that for both Q1 and Q2, there were no primary factors that dominated the assessments of visual prosthesis approaches by the subjects, but, rather, a large handful of factors that were of comparable importance. For Q1, with 24 total input factors, the ten most influential were, in order, Important Function: *face perception* (*m*_*R*_ = 0.33), Motivations: *better care* (*m*_*R*_ = 0.40), Informed About Treatments (*m*_*R*_ = 0.42), Advice Sources: *blind community* (*m*_*R*_ = 0.44), Motivations: *risk taking/altruism* (*m*_*R*_ = 0.45), Residual Vision (*m*_*R*_ = 0.46), Important Function: *movement perception* (*m*_*R*_ = 0.46), Onset Age (*m*_*R*_ = 0.48), Education (*m*_*R*_ = 0.49), and Motivations: *therapeutic benefits* (*m*_*R*_ = 0.50).

For Q2, with 61 total input factors, the ten most influential were Important Function: *light perception* (*m*_*R*_ = 0.39), Motivations: *trust researcher* (*m*_*R*_ = 0.40), Choice Reasons: *experimental procedures* (*m*_*R*_ = 0.40), Motivations: *help research* (*m*_*R*_ = 0.40), Concerns: *risks* (*m*_*R*_ = 0.42), Concerns: *surgery* (*m*_*R*_ = 0.42), Residual Vision (*m*_*R*_ = 0.43), Information Sources: *services for the blind* (*m*_*R*_ = 0.43), Aversions: *lack of benefit* (*m*_*R*_ = 0.43), and Choice Reasons: *altruism* (*m*_*R*_ = 0.44).

## Discussion

In order to understand the acceptance of visual prostheses as a treatment for blindness, we conducted two types of questionnaires aiming to extract the perspectives of blind individuals that fit the profile a potential patient. Answers were sought regarding the themes of retinal, thalamic and cortical approaches, and our analysis included overall assessments of artificial vision and the characteristics of potential patients may shape and drive the decision to seek a device.

Overall, our findings were consistent with the interpretation that contemporary visual prosthesis devices do not yet rise to the level of acceptability by the intended population. This stark observation has substantial implications for active research and development programs. For none of the approaches were the benefits significantly positive, and the net benefit (benefit minus risk) was positive only for the Retinal approach, and only because the risks were perceived to be substantially lower than the other two approaches. Those results are aligned with previous reports from studies of single prosthesis approaches^18,19,21,68^. As in those previous studies, participants here want devices with high functionality and low risk, devices that would allow navigation, orientation and object recognition independently^18,19,22,23,68^.

In a related study using a questionnaire as the vehicle for collecting information^21^, it was found that 35% of blind patients were not willing to participate in a retinal prosthesis, with 44% unsure, and 21% willing to participate. For participants who were willing to participate, the three most commonly cited reasons were therapeutic in nature, possible therapeutic benefit (64%), to receive better care (46%), and better than no treatment at all (36%), followed by altruism (27%) and to receive more information (27%); leading reasons for declining participation were, in order of frequency, fear of potential side effects and risks (63%), lack of benefit or worse (58%), poor understanding of information about the trials (47%), financial issues (32%), and time concerns or inconvenience (26%). These observations parallel our findings to a great extent (Fig. 7), suggesting a universality in attitudes among potential recipients of a visual prosthesis given the disparate natures of the two subject populations (here, Greece; Xia, China).

### Adjusting research priorities

Xia and colleagues^21,22^ also discuss the high expectations of their subjects and, perhaps surprisingly, lay the onus on researchers performing clinical tests of visual prostheses to adjust patient expectations to more realistic levels. We suggest a different tack: scientists and engineers have a duty to increase the performance of their devices to meet the expectations of the target population more closely.

The idea that some vision is better than no vision may not be shared by the blind community, despite often being taken on faith by researchers. Researchers have broadly assumed that being implanted with a device that restores only crude vision would be better than being blind, whereas potential recipients have higher needs with more nuanced concerns^11^. One insightful question from the audience during the question-and-answer period following our lecture succinctly captured this dichotomy: “If we get one of these devices, will we still need a white cane?” Contemporary visual prostheses, as was answered, are not sufficiently advanced to provide independence from mobility assistance devices like white canes. We take this audience question to be indicative of a fundamental problem facing the current generation of devices that have low resolution and limited field of view: the minimum performance for a visual prosthesis to achieve widespread acceptance is higher than what is currently available. These views were cemented by the two write-in answers on Q1 regarding the Important Function for Restoration: “to have my sight the way it used to be,” and “to be able to go around on my own.” Similar desires of a return to high visual function were reported in Anderson, Warren, and Lee’s study of potential cortical prosthesis recipients in Australia^23^, again suggesting a universality of themes observed here.

It should also be of some concern to researchers that the level of surgical risk involved for each approach was reflected in subjects’ assessment of risk and overall acceptance. Combined with the expectation of high functional restoration, the level of potential benefit will need to be substantially increased for cortical approaches in particular. When considering, then, the balance between benefit and risk, researchers must consider real-life functionality^71^ so that patients may be promised ample benefits to counter the risks of implantation.

### Influential factors

Our analysis of which factors had the highest frequency of significant correlations with measured opinions before and after the focus groups reinforces the importance of meeting patient expectations for high levels of restored vision, building trust with patients and keeping them fully informed. These observations confirm similar themes that have been found by other studies^19,21,23^. In particular, willingness to accept treatment was highest closest to the loss of vision, and highest among younger participants here.

Beyond these two primary factors, additional factors that had broad influence were functional or therapeutic in nature, supporting various visual abilities such as recognizing faces or obtaining health care from trusted sources. Additional factors were being informed about treatments including from peer sources, understanding treatment risks, and a willingness to accept risk or being motivated by altruism. Socioeconomic factors, such as distance from treatment or compensation were not found to have as much influence.

### Future investigation

Lane, Nitsch, and Scherer^68^ observed that individuals with life-long disabilities are highly capable when providing important information and can conduct crucial health-care decisions as they gain expertise in their daily needs, as compared to researchers who may lack personal experience of living with blindness. With that in mind, we conclude that in order to make artificial vision devices useful for the target group, we must thoroughly examine patient expectations, attitudes and psychological state during selection and pre-operation procedures. As a next step to elucidating such details, reviewing open discussions with patients in focus group settings will shed more light on their thoughts.

## Conclusion

In assessing the expectations and attitudes towards visual prostheses of blind individuals who were potential recipients of these vision-restoring devices, we found a largely negative landscape due primarily to the technological limitations of current devices. Attitudes generally tracked the pathway from retina to thalamus to cortex, being most positive for the retinal approach and most negative for the cortical approach. Participant age and number of years of blindness were strong predictors of attitude toward hypothetical treatment with a visual prosthesis, with younger and more recently blind individuals being more accepting. Concerns were expressed over the level of restored function, and issues of risk and trust, suggesting that good communication between research team or physician and potential patient is a critical aspect to patient recruitment. We conclude that substantial advances are still required before broad acceptance of visual prostheses will be found.

## Methods

### Recruitment, sample and timeline of data collection

Participants were recruited through the Lighthouse for the Blind of Greece. Individuals were approached using two methods: general announcements through social media, and personal invitations to regular members of the Lighthouse. The target sample was intended to share characteristics with not only focus group participants from previous reports in the literature, but also with what is currently considered a good candidate for visual prosthesis devices. Participants were expected to have experienced significant loss of vision occurring after the age of 15–16, to be Greek speaking, and to be free of brain injury, or other major physical, psychological, or mental disorder. These criteria were verbally verified during Q1 administration, but no clinical testing or screening was performed.

#### Data collection

All aspects of presentation, data collection, and subject interaction were conducted in Greek. We first gave a lecture-style presentation at the facilities of *Faros Tyflon* of the three approaches in artificial vision, retinal, thalamic and cortical (see Supplementary Appendix S1), followed by an extended question and answer period. The event lasted about 2–1/2 h, most of which was the audience interaction.

Members of the audience were then invited to fill out *Questionnaire 1: Short* (Q1; see Supplementary Table S1, Supplementary Fig. S1﻿). Q1 included questions about demographics, age, cause and progress of blindness and simple multiple-choice questions about benefits and perceived risk of the three approaches, and consultation about medical decision making. Each participant required 5 to 7 min to complete a questionnaire with the assistance of a member of the study staff who presented each question in turn with possible answers and recorded the participant responses.

From these responses, we screened subjects into seven focus groups with two to five individuals per group. Each focus group was co-lead by a pair of interviewers (authors VK, EP). Focus group sessions began with a review of the lecture. The interviewers then guided the discussion through a list of predermined topics and specific questions. Main themes that were explored included experience with blindness, information that subjects felt would be necessary before making a decision on receiving an implant, general attitudes towards artificial vision treatments, and specific attitudes towards particular approaches (Supplementary Table S2). Audio recordings were made of each group for separate analysis (not reported here). Focus groups typically lasted 2 h. Participants were compensated for their time.

After the focus groups had concluded, participants completed the *Questionnaire 2: Long* (Q2; see Supplementary Table S3) through individual interviews by members of the study staff, either in person or by telephone. As with Q1, interviewers presented each question with possible answers, and recorded the participants’ responses. No participant elected the option of a braille questionnaire. Q2 was thematically similar to Q1, but included more demographic details and more questions with regard to details of each prosthesis approach and the decision process.

### Analysis

Quantitative analyses were used to assess relative frequencies from the questionnaires. Remaining vision was converted to a normalized scale (given fractions were retained; movement perception became 0.05; perception of light, 0.02). Distance to residence was calculated for road travel from the residence town or neighborhood using Google Maps to Syntagma Square at the center of Athens, applying a minimum value of 1 km (straight line travel was used for the one island resident). Five-level Likert responses were converted to numeric scores with the most negative (“– –” in Figs. 1, 2, 3, 5) being –2, less negative (“–”) –1, neutral (“o”) 0, slightly positive (“+”) +1, and the most positive (“++”) being +2. For analyses of the two questionnaires, we used SPSS and MATLAB to extract differences and relationships between variables and to discern any changes between the initial and final questionnaires. Unless stated otherwise, *p* = 0.05 was used as the threshold for statistical significance. Statistical *p* values are reported to the first significant digit (rounded up), with values smaller than 0.0005 reported as 0.000.

### Ethics statement

The research protocol used in this study was approved by the Institute Review Board of the Massachusetts General Hospital, and the Ethics Committee of the Department of History and Philosophy of Science at the University of Athens. It adhered to the guidelines of the Declaration of Helsinki. Informed consent was obtained from each of the participants.

## Supplementary Information

Supplementary Information.

## Data Availability

The data collected for this study are available upon reasonable request to the communicating author (JSP).
