# Supplementary material for: Attitudes of potential recipients toward emerging visual prosthesis technologies
Source: Sci Rep. 2023 Jul 6;13:10963. doi: 10.1038/s41598-023-36913-8 (PMC10325978; doi:10.1038/s41598-023-36913-8)
Supplement: Supplementary file 1 — Supplementary Information. [file 41598_2023_36913_MOESM1_ESM.pdf]

SUPPLEMENTARY MATERIAL

Attitudes of potential recipients toward emerging visual prosthesis technologies

Vicky Karadima, Elizabeth Pezaris, John Pezaris

Scientific Reports, 2023

Appendix S1: Script for Presentation to *Faros Tyflon* S2
Table S1: Questionnaire 1 . . . . S11
Table S2: Focus Group Interview Structure S13
Table S3: Questionnaire 2 . . . . S15
Figure S1: Example Completed Questionnaire 1 . . . . S18
Figure S2: Accept / Reject (Q2) S19
Figure S3: General Attitude (Q2) S20

Appendix 1: Script for Presentation to *Faros Tyflon*

Written by John S. Pezaris
Translated to Greek and Delivered by Vicky Karadima

1. TITLE SLIDE

Good afternoon. Thank you all for coming today. I have been working for over a decade at Harvard Medical School in order to be able to speak with you. I must tell you that I am a scientist and not a doctor. If you have medical questions, I will not be able to answer them.

Today, I want to talk to you about the efforts in my laboratory to create a prosthetic device that will provide artificial sight. Also, I want to ask you to help with the research that my student Ms. Karadima will conduct about blindness and devices that could provide sight.

2. FOCUS GROUPS

Ms. Karadima is using focus groups to help understand factors that contribute to the participation of blind people in experimental vision restoration procedures. We want your views and opinions, to hear about your experience with blindness and low vision. We will use your responses to search for factors that can influence the decision to receive an implant, and how patients think about the experimental process. Importantly. We use your answers to understand the needs of people with blindness when it comes to such therapeutic methods.

3. THE PROBLEM: BLINDNESS

Blindness is widespread, and in many millions of people. For this talk, we will concentrate on the sort of blindness that means you cannot see at all, that you can't walk without the risk of falling, or you can't see the food on your plate.

Blindness has consequences for the individual, the family, and the economy. In the US, where we are now approaching full employment in the general population, unemployment among the blind is 75%. Blindness places significant burdens on the family, both medically and in daily care. The total cost of blindness to American society is three billion dollars each year.

4. THE CAUSES: DISEASES OF THE EYE

Most of the causes of blindness are diseases of the eye. To me, as a neuroscientist and engineer, this is interesting because it only affects the eyes and not the rest of the brain. We'll see why this is important in a minute.

5. GLAUCOMA, RETINITIS PIGMENTOSA, MACULAR DEGENERATION

The most common causes of blindness are glaucoma, retinitis pigmentosa, and macular degeneration. There are many others, such as different types of cancer, but these three are the biggest.

An important fourth cause, although less common, is trauma. The trauma may be from a car or motorcycle accident, an explosion, or a bullet.

6. INFECTION, ETC.

In the developing world, infection and cataracts are the leading causes of blindness, but I won't talk about their treatment because antibiotics and lens replacement, respectively, are easy and often inexpensive ways to prevent blindness or restore vision.

Similarly, blindness that strikes either before birth or shortly thereafter leaves the visual part of the brain without the experience needed for development. Therefore, at least for today, we will not talk about the treatment of these individuals.

7. A SOLUTION: CAMERA-TO-BRAIN

So, what is the solution? When the eyes are no longer sensitive to light, as neuroscientists, we have discovered that the rest of the brain is mostly fine. And as engineers, we know how to build cameras to record video, so perhaps we can just plug information from cameras straight into the brain and have vision.

8. SIGHT 2 BLIND IMAGE

The basic idea is just that. We put a small camera, like the one in a cell phone, on a set of glasses. Video from the camera is delivered wirelessly to electrodes implanted in the brain and this provides visual restoration or artificial vision. We know how to make tiny cameras. We know how to build small computers. We know how to make wireless connections. We know how to make brain electrodes.

9. SOUNDS SIMPLE, RIGHT?

It sounds very simple, but that's the idea. The hard part is translating the camera images into the language of the brain, the neural code, and delivering the translation in a safe way to create images that the brain can understand.

10. VENTRAL VIEW OF HUMAN BRAIN

Here is a drawing of the human brain showing the early visual pathway, which starts from the eyes and ends in the brain. Let me describe how it works.

Light enters the eyes where it is transformed into neural activity. Most causes of blindness are diseases that interfere with this process, making the eye no longer sensitive to light.

But normally, nerve signals continue to the optic nerve and lateral geniculate nucleus (or LGN for short) and from there via the optic radiation to the primary visual cortex at the back of the skull where conscious vision begins. From V1, as it's called, signals travel to different parts of the brain for things like recognizing objects, people, and places. About 40% of the human brain is dedicated to vision, mostly in areas that are not shown in this diagram.

To restore vision when eyes are not working properly or are missing, we can think of placing signals from an electronic camera at any stage of the early visual pathway. Each stage uses a slightly different way to represent images, and has a different encoding. We understand those encodings thanks to many decades of research.

11. EVIDENCE

There is evidence starting nearly 100 years ago for the creation of visual perceptions by electrical stimulation of the early visual pathway. There are reports from placing electrodes in the eyes, optic nerve, LGN, primary visual cortex, and superior visual areas.

12. BRINDLEY, LEWIN (1968) X-RAY

Perhaps the first major attempt at a visual prosthesis was by Brindley and Lewin at the University of Cambridge in the late 1960s, where they placed 80 electrodes on the surface of the primary visual cortex, Area V1 that I pointed out before. Their work was remarkable biomedical engineering that was well ahead of its time. They were able to create small points of light, either small stars or fuzzy circles, which we now call *phosphenes*.

Phosphenes are visual perceptions that occur without visual input. If you are sighted, you perceive phosphenes when you close your eyes before sleep---those purple or reddish waves. You perceive them when you press your eyes with your fingers. Or if you hit your head really hard---in both English and Greek, we say that we "see stars." These are all examples of phosphenes. Even astronauts report phosphenes when cosmic rays hit their eyes.

Naturally occurring phosphenes in blind people have not been studied as much as they should have. We would love to hear about visual experiences that blind audience members have had.

13. BRINDLEY, LEWIN (1968) PHOSPHENE LAYOUT

I am now showing a map of the phosphenes from each electrode that appeared to Brindley and Lewin's patient within their field of view. There are 78 fuzzy spots that are along a strip of the field of view, like a strip of the night sky. Each of the 78 fuzzy points created can be turned on or off. Although Brindley and Lewin did not produce more than one phosphene at a time, if there were more of them, you can imagine rough designs being created with groups of five, six, ten or twenty, just as the ancient Greeks imagined the stars to form constellations among the stars which were close one to another.

So we believe we can create the same sort of drawings using phosphenes---the pixels of artificial vision---by applying electrical stimulation to the early visual system. And with enough phosphenes, we think we can make comprehensible images.

14. WORK THUS FAR

Globally, there are approximately 24 independent groups working to create visual prosthesis. Each takes a slightly different approach.

15. OVERALL VIEW OF PROJECTS

Many of them put electrodes in the eye to stimulate any remaining healthy retinal cells. Some put electrodes on the optic nerve. Some, like my lab, put electrodes in the LGN. And some put electrodes in the visual cortex.

But they all more or less follow the same idea: using an artificial imaging system to apply patterns of electrical stimulation to restore vision.

They also, more or less, have the same big problem: the visual system is amazing, taking in tremendous, detailed information to produce a rich experience. The problem is that no group is talking about restoring normal vision. No one. Everyone is working to create a crude form of vision that we think is better than blindness, but, for now, it's very, very crude. Some teams, like mine, aim for the highest quality artificial vision, which is taking more time. While some teams instead work to make a device available as quickly as possible, so they use a lower resolution. The hard part is not the technological design of the external device --- the camera, the video processor, the wireless connection and the stimulator --- but the electrodes themselves, making everything biocompatible and ensuring that all the components work smoothly.

16. THE IMPORTANCE OF SIMULATIONS

Since implanting electrodes in a human is a tricky thing to do, many teams rely on artificial vision simulations to answer questions about how well the design they are developing will

work. They use virtual reality techniques and sighted people to do this. For people in the audience who can see, I want to show you an example from my laboratory.

17. SIMULATION VIDEOS

This is a simulation I created for this exact purpose. On the left is a black and white video of a young woman from an ordinary camera. On the right is an artificial vision simulation that has 500 phosphenes. Unfortunately, the video will only make sense to the sighted audience members, but I'll try to describe the images for everyone as best I can.

Notice a few things. First, the 500 phosphenes are not all side by side, like the pixels on your computer screen or cell phone. They are separated, as in the map by Brindley and Lewin, although there are more now. They are separated because we cannot make very thin wires and implant so many that the phosphenes become continuous. The upper limit is probably a few thousand wires, because we have to make sure that the brain tissue that the wires enter is not affected too much. If we used much more, they would cause damage and that part of the brain would probably die. So we have to be smart about biocompatibility to maximize the number of phosphenes we have, as each electrode creates one photopsie.

Then, notice that if I only showed you the right image, you wouldn't have understood that it is a woman's face. Watch carefully as I begin the video.

The brain does a wonderful job of gathering information and soon you can see that the picture through the phosphenes. You can see the woman. You can see her looking at the camera, smiling. Maybe you might recognize her if you know her, and maybe you could tell it's a bright day as she raises her arms against the sun.

The left image is a normal video with about 500,000 pixels and the one on the right only about 500 phosphenes. 500 phosphenes will not give perfect vision. Not by any stretch of the imagination, but it's not that bad. It would certainly be useful sight.

18. DIFFERENT APPROACHES

Let me describe some of the projects that have been done around the world. I will focus on the three different approaches that have received the most attention from researchers: the retinal approach, the thalamic approach, and the cortical approach. If you remember where I told you about the early visual pathway, these three approaches correspond to the three major stations along that pathway.

19. RETINAL PROSTHESIS

The general advantage of placing stimulating electrodes in the eye is that the surgical intervention is relatively limited.

However, there are some disadvantages. The most important part of the field of view is right in the center. This is the part of the eye used for details. When we look at something, we point our eyes in order to gaze at that thing. This part of the eye, in the center, is called the fovea. It is very distinct and made very differently from the rest of the eye. Unfortunately, because of these differences, we do not currently believe that it will be possible to achieve high-resolution vision with implants in the eye.

There are two different products approved for implantation in the eye for people with retinitis pigmentosa. Because of differences in the health of the eye, other diseases are not currently treated with a retinal approach. The two devices are Alpha AMS by Retina Implant AG in Germany, the Argus II from Second Sight in the USA. Neither is available right now in Greece.

The two devices are similar, but they have differences. Both work by applying small amounts of electricity to the blind part of the eye to create patterns of phosphenes that can help vision. Among the differences is that the Argus II puts a series of electrodes on the eye and uses an external camera, and the other, Alpha AMS, places the chip of the camera itself in the eye.

20. ARGUS II

The Argus II has an array of 60 electrodes that is implanted in the eye, on the retina. It uses glasses with a built-in small camera and an external control element called a Video Processing Unit. There are about 200 people who have Argus II device worldwide.

21. ALPHA AMS

The Alpha AMS places the camera chip that is combined with stimulating electrodes in the eye, just below the retina. It has no external glasses, but has an external control unit like the Argus II.

22. THALAMIC APPROACH

The next type of device I will describe is the one being developed in my lab. We are working with two industrial partners to create a brain implant that will enable the treatment of a wide range of diseases that cause blindness. Specifically, it will treat blindness from glaucoma, the most common cause of blindness. We do not have a device, yet.

23. SCHEMATIC DESIGN OF THALAMIC PROSTHESIS

We are still about five years away from having a device ready for use in humans. Electrodes are placed in the LGN, near the center of the brain, and connected to the stimulator. Glasses with digital cameras communicate wirelessly with the electrodes, providing visual representations.

The surgery is done just like for placing electrodes in a nearby part of the brain for Parkinson's disease. There are nearly 150,000 people with Parkinson's implants currently worldwide which are safe and effective. We will use very similar implant materials and techniques.

24. CORTICAL PROSTHESIS

The third main approach is to place electrodes in the primary visual cortex, at the back of the head. There are currently no devices approved devices for this approach, although Second Sight is testing the Orion system in the US. It is analogous to the design for their Argus II that we just talked about.

25. ORION

This design also uses glasses with a built-in camera. There is an external processing unit here too. There is an implanted stimulator here too.

The electrodes now go directly into the brain, and the part of the brain they contact is somewhat larger, so the skull has to be opened for implantation.

One of the problems is that the most important central part of the visual field in the primary visual cortex lies between the two brain hemispheres, so the electrodes must be applied between the hemispheres.

Another problem with the approach is that the brain is not smooth, but has many folds. The visual field is mapped to the surface of the cortex, and the electrodes only touch the outer part, so there are parts of the visual field that will not be represented, even with many, many electrodes. Also, we do not know if cortical implants will ever be able to provide color. However, a cortical implant should work for the same wide ranges of diseases as a thalamic implant.

26. REVIEW

Now let me give a quick summary of each of the approaches, citing advangates and disadvantages, giving the main points of each.

27. RETINA

First, we mentioned the retinal approach. This eye implant is intended for people with retinitis pigmentosa, or maybe macular degeneration. Eye surgery is required. The approach may be useful for those with partial blindness. The resolution of the existing implants is very low. We talked about two devices, the Argus II and the Alpha AMS. The Argus II has only 60 phosphenes, but it is available now. The Alpha AMS integrates the camera into the eye implant, and does not have external glasses.

28. LGN

Then we talked about the thalamic approach. This approach can cover all causes of blindness from eye diseases or injuries. Brain surgery is again required, but it is minimally invasive. The approach uses techniques like those that are already approved for Parkinson's disease. We may be able to include central vision which provides details of sight. It should have good resolution, maybe even very good. That is, it will have many phosphenes. It will probably be available for human use in about five years. Maybe it will have color. Digital camera glasses are still required. It's probably only for total blindness.

29. CORTICAL APPROACH

Last, we talked about the cortical approach. These devices will also be able to cover all causes of blindness from eye diseases or injuries. Again, brain surgery is required, which currently is quite invasive. The current devices have low resolution, but maybe in the future they will provide good resolution. We expect approved devices in 2 or 3 years. It is currently without color, and again has digital camera glasses. Finally, it is probably only for people with total blindness.

30. FOCUS GROUPS

If you are interested in helping us shape the future of visual prostheses, fill out one of our forms to join a focus group. We will ask you to sit down with my colleagues, Vicky and Betty, while we record your conversations about what you heard today, including any questions or concerns that remain unanswered. We will also ask you to complete two questionnaires, one before and one after. The information you give us will be compiled and put into a report that we will publish and help guide the development of our device, as well as that of other groups.

Please understand that we are not currently recruiting for implants, and that your choice of participating or will not affect whether you will have a chance to get treatment in the future.

31. SIGHT TO BLIND

Thank you for listening to our presentation.

Table S1: Questionnaire 1 (Translated)

1	Age	*(number of years)*
2	Sex	*(free-form answer)*
3	Residence	*(free-form answer)*
4	Education	*(pick one option)*<br>*a. elementary*<br>*b. secondary*<br>*c. tertiary*<br>*d. college*<br>*e. graduate school*
5	Cause of blindness	*(free-form answer)*
6	Type of impairment	*a. total*<br>*b. partial*
7	Level of residual vision	*(free-form answer)*
8	Age of onset	*(number of years)*
9	What is your level of involvement in the community (concerning impairment, support, clinical care, treatment options)?	*Likert scale**
10	How informed are you about choices for therapy and vision restoration?	*Likert scale**
11	Which function is the most important for life in Greece?	*(pick one option)*<br>*a. light perception*<br>*b. motion*<br>*c. face perception*<br>*d. light localization*
12	Would you be willing to participate in Artificial Vision clinical testing?	*a. yes*<br>*b. no*<br>*c. unsure*
13	What would motivate you to participate in an Artificial Vision project?	*(pick as many as are relevant)*<br>*a. therapeutic benefits*<br>*b. advance research*<br>*c. help other patients*<br>*d. learning*<br>*e. trust researcher/institution*<br>*f. better than no treatment*

		g. better attention or care<br>*h. monetary compensation*<br>*i. willing to take risk*
14	Whom would you consult about participating?	*(pick as many as are relevant)*<br>*a. family*<br>*b. friends*<br>*c. physician*<br>*d. psychologist*<br>*e. community members*
15	Rate retinal approach for risk	*Likert scale**
16	Rate thalamic approach for risk	*Likert scale**
17	Rate cortical approach for risk	*Likert scale**
18	Rate retinal for benefits	*Likert scale**
19	Rate thalamic approach for benefits	*Likert scale**
20	Rate cortical approach for benefits	*Likert scale**
21	Rate retinal approach for accepting potential participation	*Likert scale**
22	Rate thalamic approach for accepting potential participation	*Likert scale**
23	Rate cortical approach for accepting potential participation	*Likert scale**
24	Rate retinal approach for declining potential participation	*Likert scale**
25	Rate thalamic approach for declining potential participation	*Likert scale**
26	Rate cortical approach for declining potential participation	*Likert scale**

Table S1: Questionnaire 1. Likert scales were presented in various forms, such as “a very small amount; a small amount; neutral; a large amount; a very large amount” or “strongly disagree; disagree; neither agree nor disagree; agree; strongly agree”; or “strongly reject; reject; neither accept nor reject; accept; strongly accept”, all of which were translated to an ordinal scale for analysis as double-minus (– –), minus (–), zero (o), plus (+), double-plus (+ +), and from there to a numeric scale of –2 to +2.

Table S2: Focus Group Interview Structure

Interview theme	*Notes*
**Welcome/Opening**	
**Brief review of Artificial Vision approaches**	
**Blindness experience**	
Describe your experience with your impairment.	*How visual impairment has negatively or positively affected your life, challenges and lesson?*
Is there any special aspect concerning experience with your impairment, while living in Greece?	
What is your relationship with /participation rate in the blind community /blindness institutions?	
Are you getting informed about treatment, support etc. for your condition?	
**General attitudes for Artificial Vision**	
What were your reactions upon learning about the…<br>…retinal projects?<br>…thalamic project?<br>…cortical projects?	*Have you heard about this or similar approaches before?*
What were your reactions upon learning about Artificial Vision?	
What was your understanding of the risks of each project?	
What was your understanding of the benefits of each project?	
**General attitudes on participation in Artificial Vision clinical trials**	
What are your concerns about Artificial Vision trials?	*Fears, aspects disliked?*
What, if any, expectations would you have about such a procedure and the outcome?	*Expected gains*
**Opinion for different Artificial Vision approaches**	
What was appealing about participating in the preferred approach?	*Or in any approach you would like to comment*
What were your concerns about the preferred project?	
What were the declining reasons for the other approaches?/ How did you go about deciding to participate in the project?	*Decline or accepting a project*
What would motivate you to participate in the preferred project?	

**Information**	
In what ways the information session and informed consent impact the decision?	
Is there anything you would like to have known before deciding to participate?	
What should we, as a research team, do to make this a good experience for the volunteers for a new visual device?	
What characteristics the ideal candidate for Artificial Vision trial should have?	
What is the level of involvement a candidate should have in experimental trials?	*What is the preferred level of involvement?*
**Questions**	
What kind of information was important to you while you were making a decision?	
What are your questions about each approach?	
**Closing remarks**	
Is there anything you would like to have known before deciding to participate?	
What should we as a research team do to make this a good experience for the volunteers for a new visual device?	

Table S2: Focus Group Interview Structure. Each focus group was lead using the structured interview questions listed above, allowing subjects to freely respond in the group setting.

Table S3: Questionnaire 2 (Translated)

1	Age	*(number of years)*
2	Sex	*(free-form answer)*
3	Residence	*(free-form answer)*
4	Education	*(pick one option)*<br>*a. elementary*<br>*b. secondary*<br>*c. tertiary*<br>*d. college*<br>*e. graduate school*
5	Cause of blindness	*(free-form answer)*
6	Type of impairment	*a. total*<br>*b. partial*
7	Level of residual vision	*(free-form answer)*
8	Age of onset	*(number of years)*
9	Level of involvement in the community (concerning impairment, support, clinical care, treatment options)	*Likert scale**
10	What are your sources of information for the aspects mentioned in Q9?	*(pick as many as are relevant)*<br>*a. internet*<br>*b. telephone (Voicemail)*<br>*c. television / radio*<br>*d. Social Interaction*<br>*e. Institutions for the blind*<br>*f. other*
11	What is the level of information about treatment and restoration options?	*Likert scale**
12	What function is the most important, while living in Greece?	*(select most important option)*<br>*a. light perception*<br>*b. face perception*<br>*c. movement perception*<br>*d. light localization*<br>*e. activities of daily living*
13	Would you be willing in participating in Artificial Vision clinical implantation and testing?	*a. yes*<br>*b. no*<br>*c. unsure*
14	What would motivate you to participate in an Artificial Vision project?	*(select one or more)*<br>*a. therapeutic benefits*<br>*b. help/advance research*<br>*c. help other patients*<br>*d. learning*

		e. trust in researcher/institution<br>*f. better than no treatment*<br>*g. better attention or care*<br>*h. payment/compensation*<br>*i. willing to take risk*<br>*j. other [open]*
15	Why would you decline to participate?	*(select one or more)*<br>*a. fear of risks and side effects*<br>*b. no therapeutic benefits*<br>*c. worse vision*<br>*d. poor understanding of processes and outcomes*<br>*e. aversion of experimental procedure*<br>*f. lack of time*<br>*g. lack of financial support or lack of compensation*<br>*h. research site and transportation*<br>*i. other [open]*
16	What concerns could influence your decision?	*(select one or more)*<br>*a. surgery*<br>*b. risks and side effects*<br>*c. outcomes*<br>*d. understanding of processes*<br>*e. experimental procedures*<br>*f. time needed*<br>*g. payment*<br>*h. location and transportation*<br>*i. information revealed through testing process*<br>*j. communication with researchers or institution*<br>*k. other [open]*
17	Whom would you consult about participating?	*(select one or more)*<br>*a. family*<br>*b. friends*<br>*c. physician*<br>*d. psychologist*<br>*e. community members*<br>*f. other [open]*
18	Which of the approaches presented would you most likely choose to participate?	*(select one)*<br>*a. retinal*<br>*b. thalamic*<br>*c. cortical*
19	What are your main reasons for this choice?	select reasons for Questions 13-15<br>*a. general therapeutic benefits*<br>*b. visual perception outcomes*<br>*c. to help research*<br>*d. to help future patients/altruism*<br>*e. educational purposes/learning process*<br>*f. trust in the researcher/research structure g. communication with researchers or institutions*<br>*h. better care*

		i. better than non-treatment<br>*j. payment/compensation*<br>*k. risk willingness*<br>*l. time*<br>*m. surgery*<br>*n. experimental procedures*<br>*o. understanding of procedures*
20	Why is it important for you to follow the testing phase of visual prostheses?	*a. payment*<br>*b. outcomes*<br>*c. support*<br>*d. information provided*<br>*e. team communication*<br>*f. feedback*
21	Rate the retinal approach for risk	*Likert scale**
22	Rate the thalamic approach for risk	*Likert scale**
23	Rate the cortical approach for risk	*Likert scale**
24	Rate the retinal for benefits	*Likert scale**
25	Rate the thalamic approach for benefits	*Likert scale**
26	Rate the cortical approach for benefits	*Likert scale**
27	Rate the retinal approach for accepting potential participation	*Likert scale**
28	Rate the thalamic approach for accepting potential participation	*Likert scale**
29	Rate the cortical approach for accepting potential participation	*Likert scale**
30	Rate the retinal approach for declining potential participation	*Likert scale**
31	Rate the thalamic approach for declining potential participation	*Likert scale**
32	Rate the cortical approach for declining potential participation	*Likert scale**
33	Rate the retinal approach for general impression	*Likert scale**
34	Rate the thalamic approach for general impression	*Likert scale**
35	Rate the cortical approach for general impression	*Likert scale**

Table S3: Questionnaire 2. Likert scales were presented here as for Q1 and translated to first an ordinal scale and then to a numeric scale for analysis, as described in the legend for Q1.

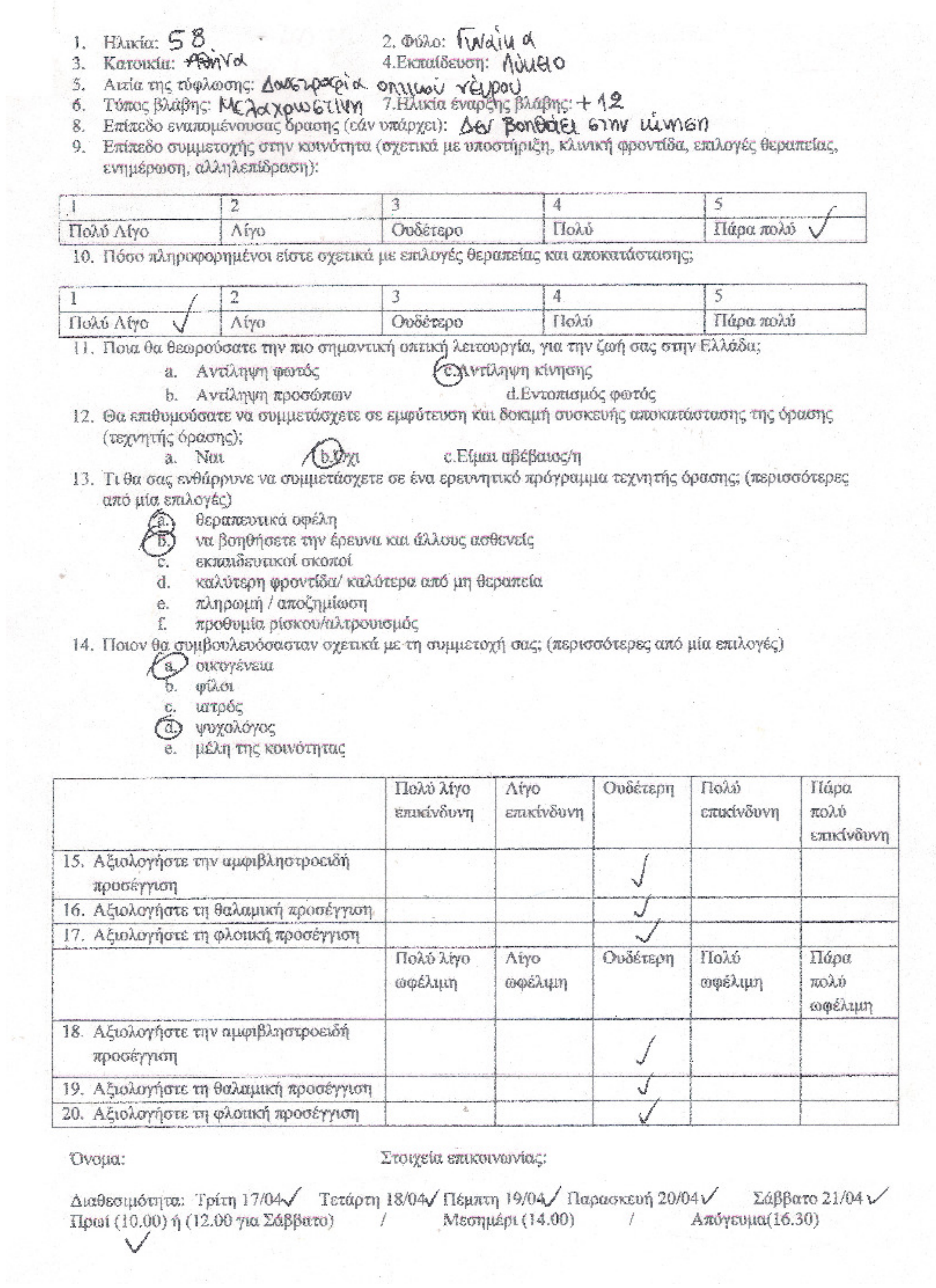

1. Ηλικία: 58 2. Φύλο: Γυναίκα
3. Κατοικία: Αθήνα 4. Εκπαίδευση: Λύκειο
5. Αιτία της τύφλωσης: Δυστροφία οπτικού νεύρου
6. Τύπος βλάβης: Μελαχρωστίνη 7. Ηλικία έναρξης βλάβης: +12
8. Επίπεδο εναπομένουσας όρασης (εάν υπάρχει): Δεν βοηθάει στην κίνηση
9. Επίπεδο συμμετοχής στην κοινότητα (σχετικά με υποστήριξη, κλινική φροντίδα, επιλογές θεραπείας, ενημέρωση, αλληλεπίδραση):

1	2	3	4	5
Πολύ Λίγο	Λίγο	Ουδέτερο	Πολύ	Πάρα πολύ ✓

10. Πόσο πληροφορημένοι είστε σχετικά με επιλογές θεραπείας και αποκατάστασης;

1	2	3	4	5
Πολύ Λίγο ✓	Λίγο	Ουδέτερο	Πολύ	Πάρα πολύ

11. Ποια θα θεωρούσατε την πιο σημαντική οπτική λειτουργία, για την ζωή σας στην Ελλάδα;
    a. Αντίληψη φωτός (c.) Αντίληψη κίνησης
    b. Αντίληψη προσώπων d. Εντοπισμός φωτός
12. Θα επιθυμούσατε να συμμετάσχετε σε εμφύτευση και δοκιμή συσκευής αποκατάστασης της όρασης (τεχνητής όρασης);
    a. Ναι (b.) Όχι c. Είμαι αβέβαιος/η
13. Τι θα σας ενθάρρυνε να συμμετάσχετε σε ένα ερευνητικό πρόγραμμα τεχνητής όρασης; (περισσότερες από μία επιλογές)
    (a.) θεραπευτικά οφέλη
    (b.) να βοηθήσετε την έρευνα και άλλους ασθενείς
    c. εκπαιδευτικοί σκοποί
    d. καλύτερη φροντίδα/ καλύτερα από μη θεραπεία
    e. πληρωμή / αποζημίωση
    f. προθυμία ρίσκου/αλτρουισμός
14. Ποιον θα συμβουλευόσασταν σχετικά με τη συμμετοχή σας; (περισσότερες από μία επιλογές)
    (a.) οικογένεια
    b. φίλοι
    c. ιατρός
    (d.) ψυχολόγος
    e. μέλη της κοινότητας

	Πολύ λίγο επικίνδυνη	Λίγο επικίνδυνη	Ουδέτερη	Πολύ επικίνδυνη	Πάρα πολύ επικίνδυνη
15. Αξιολογήστε την αμφιβληστροειδή προσέγγιση			✓		
16. Αξιολογήστε τη θαλαμική προσέγγιση			✓		
17. Αξιολογήστε τη φλοιική προσέγγιση			✓		
	Πολύ λίγο ωφέλιμη	Λίγο ωφέλιμη	Ουδέτερη	Πολύ ωφέλιμη	Πάρα πολύ ωφέλιμη
18. Αξιολογήστε την αμφιβληστροειδή προσέγγιση			✓		
19. Αξιολογήστε τη θαλαμική προσέγγιση			✓		
20. Αξιολογήστε τη φλοιική προσέγγιση			✓		

Όνομα: Στοιχεία επικοινωνίας:

Διαθεσιμότητα: Τρίτη 17/04 ✓ Τετάρτη 18/04 ✓ Πέμπτη 19/04 ✓ Παρασκευή 20/04 ✓ Σάββατο 21/04 ✓
Πρωί (10.00) ή (12.00 για Σάββατο) ✓ / Μεσημέρι (14.00) / Απόγευμα(16.30)

Figure S1: Example Completed Questionnaire 1. A typical completed Q1.

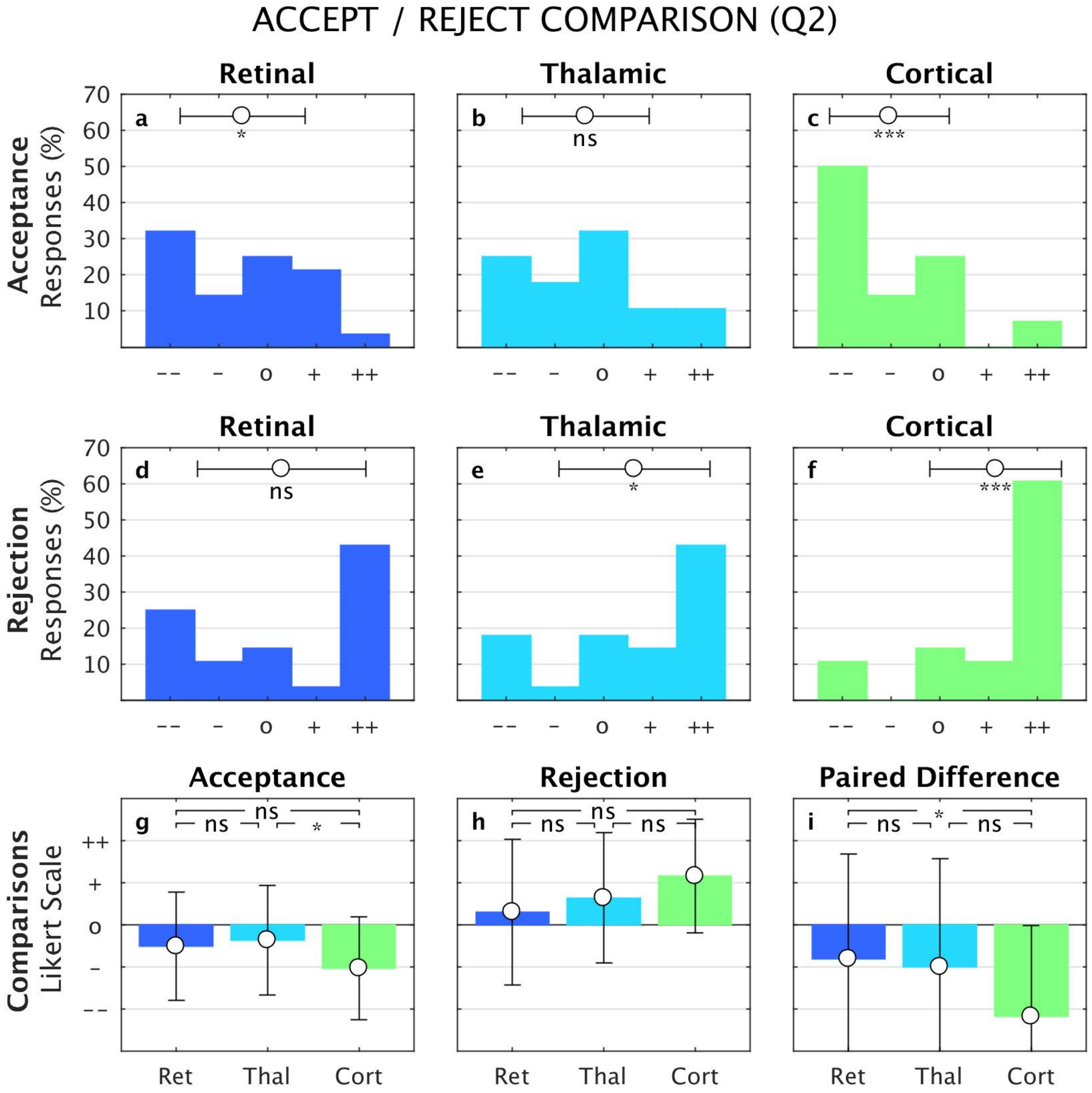

Figure S2: Accept / Reject (Q2). Distribution of Likert scale responses for Acceptance and Rejection of hypothetical treatment for the three different approaches. The distributions of Acceptance (Panels a, b, c) were all negative in the mean, with Retinal and Cortical significantly deviated from neutral. For Rejection (Panels d, e, f), the distribution for Retina appears to be bimodal, and is not significantly different from neutral, whereas for Thalamic and Cortical approaches are significantly above neutral. When comparing Acceptance across approaches (g) all are shown negative, Rejection (h) all are shown positive, with the pairwise difference (i) increasingly negative from Retinal to Thalamic to Cortical approaches.

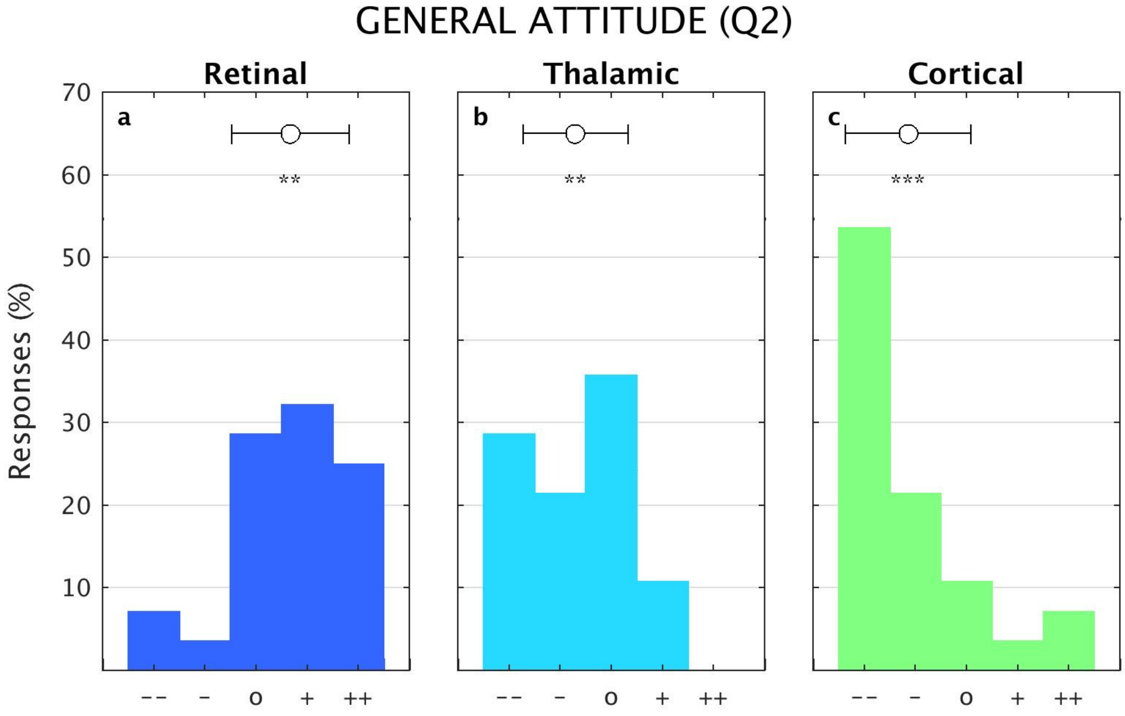

Figure S3: General Attitude (Q2 only). Distribution of Likert scale responses for General Attitude toward the three different approaches. The Retinal approach (Subpanel a) shows a significantly positive response among subjects, whereas the Thalamic (b) and Cortical (c) approaches show significantly negative responses. For all plots, $n = 27$.
